# Underestimation of Invasive Meningococcal Disease in Italy

**DOI:** 10.3201/eid2203.150928

**Published:** 2016-03

**Authors:** Chiara Azzari, Francesco Nieddu, Maria Moriondo, Giuseppe Indolfi, Clementina Canessa, Silvia Ricci, Leila Bianchi, Daniele Serranti, Giovanni Maria Poggi, Massimo Resti

**Affiliations:** Anna Meyer Children’s University Hospital, Florence (C. Azzari, F. Nieddu, M. Moriondo, G. Indolfi, C. Canessa, S. Ricci, L. Bianchi, D. Serranti, G.M. Poggi, M. Resti);; University of Florence Department of Health Sciences, Florence, Italy (C. Azzari, F. Nieddu, M. Moriondo, S. Ricci, D. Serranti, G.M. Poggi)

**Keywords:** Neisseria meningitidis, invasive meningococcal disease, underestimation, PCR, underreporting, misdiagnosis, bacteria, bacterial meningitis, Italy

## Abstract

Knowing the incidence of invasive meningococcal disease (IMD) is essential for planning appropriate vaccination policies. However, IMD may be underestimated because of misdiagnosis or insufficiently sensitive laboratory methods. Using a national molecular surveillance register, we assessed the number of cases misdiagnosed and diagnoses obtained postmortem with real-time PCR (rPCR), and we compared sensitivity of rPCR versus culture-based testing. A total of 222 IMD cases were identified: 11 (42%) of 26 fatal cases had been misdiagnosed or undiagnosed and were reclassified as IMD after rPCR showed meningococcal DNA in all available specimens taken postmortem. Of the samples tested with both rPCR and culture, 58% were diagnosed by using rPCR alone. The underestimation factor associated with the use of culture alone was 3.28. In countries such as Italy, where rPCR is in limited use, IMD incidence may be largely underestimated; thus, assessments of benefits of meningococcal vaccination may be prone to error.

## Abstract 2

Underestimation is attributable to misdiagnosis, especially in fatal cases, and use of insufficiently sensitive laboratory methods.

*Neisseria meningitidis* is the major etiologic agent of bacterial meningitis and one of the most important causes of invasive bacterial disease worldwide (*1*,*2*). The annual number of invasive meningococcal disease (IMD) cases is estimated to be at least 1.2 million, resulting in ≈135,000 deaths (*3*). Meningococcal meningitis is the most common form of meningococcal disease, accounting for 80%–85% of all reported cases of this illness. In nearly half of these cases, sepsis is also present. The remaining 15%–20% of cases are sepsis only (*1*–*3*); however, in the elderly, *N. meningitidis* can also cause pneumonia (*4*).

In Italy, recent data show that IMD in children results in a death for ≈13% (7%–8% for meningitis and 20% for sepsis) of case-patients (*5*). Among survivors, 10%–30% have disabling, long-term sequelae such as seizures, motor impairments, hydrocephalus, sensorineural hearing loss, mental retardation, and cognitive and behavioral problems (*2*,*6*). 

IMD has a high economic and social impact, and a vaccination program could be useful in reducing incidence of disease. However, to gauge the value of vaccination through the use of health technology assessment (*7*), precise data on IMD incidence are needed. Furthermore, meningococcal infection has a rapid and severe clinical progression and clinical signs and symptoms that are similar to severe invasive infections caused by other pathogens. Consequently, a fast and sensitive method of diagnosis is needed to ensure that contacts of meningococcal disease patients receive appropriate prophylaxis to prevent secondary cases. Standard diagnostic microbiology using culture-based methods is critical, enabling molecular characterization of isolates and providing information on antimicrobial drug resistance. However, culture-based methods are strictly dependent on viability of microorganisms. That characteristic may be a serious limiting factor, especially in patients who have a rapid fatal outcome or who have already undergone antimicrobial therapy (*8*). 

*N. meningitidis* is a fastidious pathogen that frequently undergoes autolysis, and its growth can be inhibited by a single dose of antimicrobial drug therapy, even in cases when the patient dies from the infection (*9*). Therefore, molecular tests such as real-time PCR (rPCR) are used alone or in combination with culture to diagnose IMD and determine the serogroup of the implicated pathogen (*5*,*10*). However, in countries where use of rPCR is limited, IMD may go undiagnosed. Failure to diagnose IMD is undermining prevention efforts and evaluation of IMD incidence and leads to underestimation of IMD and imprecise assessments of the relative risks and benefits of vaccination. By using data from Italy’s national register for molecular surveillance of invasive bacterial disease, we attempted to identify factors contributing to the underestimation of IMD, including suddenly fatal cases and the use of different diagnostic procedures.

## Methods

### Patients

Our study evaluated retrospectively all patients included in the molecular surveillance register during 2007–2014. The register was started in 2006 and has been expanded since 2007 with dedicated funds from Italy’s Center for Disease Control through a project titled “Improving Diagnosis of Invasive Bacterial Infection by Molecular Methods.” The project and, consequently, the register were initially focused on pediatric hospitals. All pediatric hospitals or pediatric wards in general hospitals in Italy were invited to participate. Upon request by clinicians, samples obtained from adults were also accepted, tested, and included in the register, and the number of adults tested has increased over the years. Molecular surveillance was organized and is still active as a voluntary, nonmandatory surveillance. To be included in the register, at least 1 sample from each patient had to be analyzed by using rPCR, whereas use of a culture-based test was not an inclusion criterion. All clinical and laboratory data were recorded.

### Sample Collection and Testing

Samples of blood, cerebrospinal fluid (CSF), or other normally sterile fluids were obtained as soon as possible (in most cases, before start of treatment) from patients in whom, on the basis of clinical signs and symptoms, invasive bacterial disease was suspected upon hospital admission. Samples were then sent for molecular testing to the reference center (Immunology and Infectious Disease Laboratory, Anna Meyer Children’s Hospital, Florence, Italy) by using a freepost parcel carrier service; samples were delivered by the following day and tested within 2 hours after arrival. A report was produced and immediately sent back (by fax or email) to the sending hospital so that clinicians had the report within 24 hours after shipment of the sample. Samples for cultures were collected and sent to local laboratories in accordance with the hospitals’ own procedures. Sepsis was clinically suspected in the presence of previously described signs and confirmed by blood tests (*11*). Meningitis was clinically suspected in the presence of a compatible clinical syndrome and abnormal chemical test results (*12*). A case was considered to be confirmed in the presence of positive microbiologic tests (culture or molecular tests). Our study evaluated all patients included in the molecular surveillance register and was approved by the Institutional Review Board at Anna Meyer Children’s University Hospital.

### Diagnostic Criteria

A diagnosis of laboratory-confirmed IMD was made if a patient’s samples were culture positive for *N. meningitidis,* rPCR positive for the *ctrA* gene, or both, as described previously (*5*). If no increase in the fluorescent signal occurred before the 40th cycle of amplification, the sample was assumed to be negative. All samples in which the *ctrA* gene was detected by rPCR were included in a serogrouping analysis. The serogroups A, B, C, W, and Y (*13*) were identified by rPCR or endpoint PCR (for serogroups W and Y) by using appropriate primers and probes (Table 1).

**Table 1:** Primers and probes used for *Neisseria meningitidis* serogrouping of isolates from samples from a national register for molecular surveillance of invasive bacterial disease, Italy, 2007–2014

Target	Gene	Forward primer	Reverse primer	Probe
*N. meningitidis*	*ctrA*	gctgcggtaggtggttcaa	ttgtcgcggatttgcaacta	FAM_cattgccacgtgtcagctgcacat_TAMRA
Serogroup A	*sacB*	cccccagcatggctagattt	agggcactttgtggcataattt	FAM_accctaaaattcaatgggtatatcacga_TAMRA
Serogroup B	*siaD* B	ttggacttggttaagctgacctaa	gttgacaacatctccattttatcttacc	FAM_ttagatatgacaaataaattgttacgtggg_TAMRA
Serogroup C	*siaD* C	agggaaccgcaacctatgc	cacaaaacgttgctcaaattttg	FAM_ccactcttagaatcatttacatacaaaccc_TAMRA
Serogroup W/Y	*siaD* W/Y	gctgataaattgttcttatggtctgaa	cggcaccagaaccaatctct	FAM_ttggaatcatgagcttttaccaaatccaaca_TAMRA
Serogroup W*	*siaD*	cagaagtgagggatttccata	cacaaccattttcattatagttactgt	
Serogroup Y*	*siaD*	ctcaaagcgaaggctttggtta	ctgaagcgttttcattataattgctaa	


*Identified by using endpoint PCR.

## Results

### Samples Received and Diagnosis of Meningococcal Infection

**Figure 1:**
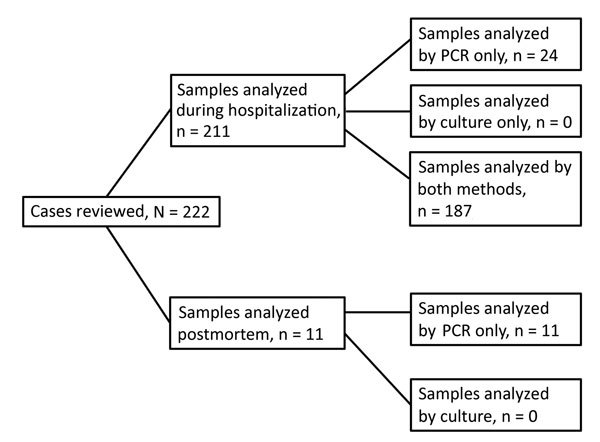
Distribution of patients diagnosed with invasive meningococcal disease during hospitalization or postmortem evaluation, by test performed for *Neisseria meningitidis* (real-time PCR [rPCR] or rPCR and culture), from a national register for molecular surveillance of invasive bacterial disease, Italy, 2007–2014.

Patients were selected from among 85 hospitals in 19 of Italy’s 20 regions. The only region that did not include any patients represents 0.2% of Italy’s population. Of 222 patients evaluated, 211 (95.0%) were tested during hospitalization and 11 (5.0%) were tested postmortem (Figure 1). At least 1 sample from each of the 222 patients included in the study was tested by rPCR. Because the reporting of a culture-based test (or lack of one) was welcome but not required for a case to be included in the register, samples for culture-based tests were not available for all patients, but at least 1 sample for culture-based tests was available for 187 of the 211 hospitalized patients. No culture-based test was performed for the 11 patients whose IMD diagnosis was postmortem; instead, diagnosis was performed by using rPCR on autoptic specimens, including blood, CSF, and formalin-fixed tissue samples (e.g., kidney, adrenal gland, brain, and lung tissue).

**Figure 2:**
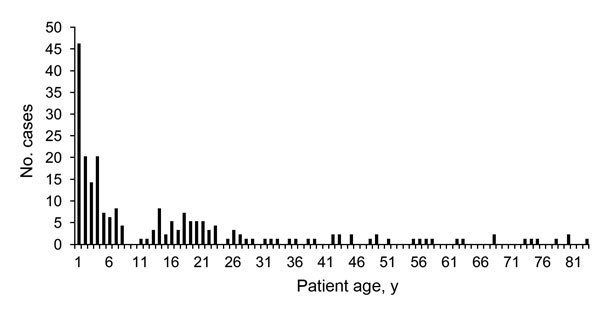
Age distribution of 222 patients diagnosed with invasive meningococcal disease, from a national register for molecular surveillance of invasive bacterial disease, Italy, 2007–2014.

Among the 222 patients with confirmed IMD, we found 171 (77.0%) meningitis cases (11 of which were associated with sepsis) and 51 (23.0%) sepsis cases. A total of 158 (71.2%) cases were found in the pediatric age group (0–18 years of age), and 64 (28.8%) cases were found in adults (>18 years of age) (Figure 2). Children <1 year of age had the highest number of cases (46/222; 20.7%). The male-to-female ratio was 121:101 (1.2).

The rPCR tests performed directly on normally sterile fluids (blood or CSF) were positive for all 222 patients, and rPCR enabled serogrouping in 218 (98.2%) cases (4 samples were not serogrouped because of insufficient sample material). Of the 218 samples that were serogrouped, 172 (78.9%) were serogroup B, 29 (13.3%) were serogroup C, 8 (3.7%) were serogroup W, and 8 (3.7%) were serogroup Y. No organisms from serogroup A were found.

During the study period (2007–2014), a total of 26 deaths occurred among the 222 patients, resulting in a case-fatality rate of 11.7%. Five (19.2%) deaths occurred in patients <1 year of age, 7 (26.9%) in patients 1–5 years of age, 8 (30.8%) in patients 6–18 years of age, and 4 (15.4%) in adult patients. Case-fatality rates were 22.6% (14/62 cases) in patients admitted with a diagnosis of sepsis or meningitis and sepsis and 7.5% (12/160 cases) in patients admitted for meningitis but with no mention of sepsis at admission.

### Misdiagnosis and Postmortem Diagnosis of IMD

Postmortem diagnosis of IMD was obtained in 11 (5.0%) of the 222 cases. In all 11 cases, culture-based tests were either negative or impossible to perform because the patient died before being admitted to the hospital. Eight of these cases had been diagnosed as sepsis of unknown origin (Table 2). Here we describe the clinical progression of the other 3 cases.

**Table 2:** Description of 11 case-patients with postmortem diagnosis of invasive meningococcal disease included in a national register for molecular surveillance of invasive bacterial disease, Italy, 2007–2014*

Patient no.	Sex	Age at death	Preexisting disease	Cause of missing or incorrect diagnosis	First diagnosis of cause of death	Culture result	rPCR result†	Serogroup
1	F	20 y	None	Died before being admitted to hospital	Sudden death	Not performed	Positive	B
2	M	5 mo	None	Died before being admitted to hospital	SIDS	Not performed	Positive	C
3	M	17 y	None	Misdiagnosis: acute myeloid leukemia	Acute myeloid leukemia	Negative	Positive	C
4	F	5 mo	None	Died before being admitted to hospital	Sepsis	Not performed	Positive	B
5	M	6 y	None	Died <1 h after hospital admission	Sepsis	Not performed	Positive	Y
6	F	11 mo	None	Died before being admitted to hospital	Sepsis	Not performed	Positive	C
7	M	4 y	None	Died before being admitted to hospital	Sepsis	Not performed	Positive	B
8	M	15 y	Previous meningitis at age 5 y	Negative culture-based tests	Sepsis	Negative	Positive	Y
9	M	20 y	Diabetes type I	Negative culture-based tests	Sepsis	Negative	Positive	C
10	M	13 y	None	Died at hospital admission	Sepsis	Not performed	Positive	C
11	M	6 y	None	Died at hospital admission	Sepsis	Not performed	Positive	B


*SIDSs, sudden infant death syndrome; rPCR, real-time PCR. †Of *ctrA* gene of *Neisseria meningitidis.*

#### Case 1

A 20-year-old, previously healthy woman had sudden onset of high fever with chills and general malaise. The next day, her general condition rapidly deteriorated. She was then referred to the emergency department but died on the way to the hospital. A diagnosis of sudden death was made; no blood test was performed. A few years later, autoptic specimens (formalin-fixed lung, kidney, and adrenal gland tissue) were tested (for legal reasons) at the Immunology and Infectious Disease Laboratory of the Anna Meyer Children’s Hospital by using rPCR; all specimens were found to be positive for *N. meningitidis* serogroup B.

#### Case 2

A 5-month-old male infant was found dead in his crib. In the preceding days, he had shown poor feeding and irritability. He was born from healthy, nonconsanguineous parents at the end of a normal pregnancy. In accordance with the national diagnostic protocol for sudden infant death syndrome (SIDS), an autopsy was performed. Autoptic samples (i.e., formalin-fixed lung, kidney, brain, and adrenal gland tissue) for diagnosis of infectious diseases were immediately transferred to the Immunology and Infectious Disease Laboratory, where rPCR showed the presence of *N. meningitidis* serogroup C in all the specimens.

#### Case 3

A 17-year-old male adolescent was admitted to the hospital with fever, diarrhea, vomiting, purpuric rash, and lethargy, symptoms that had manifested suddenly during the preceding 6 hours. He had a normal clinical history and a normal history of school attendance, and he had participated in sports. Blood tests performed on his arrival showed a high leukocyte count (>70,000 cells/μL) and a low platelet count (<38,000/μL). He died in the hospital 1 hour after his arrival. During the following days, all culture-based test results were negative, and a diagnosis of acute myeloid leukemia resulting in death was made. Three days after his death, a family member was admitted to the hospital with a similar clinical signs and symptoms. The pathologist in charge of postmortem examination for the first patient was immediately alerted so that an infectious disease diagnosis could be considered. The pathologist decided to send formalin-fixed tissue samples to the Immunology and Infectious Disease Laboratory. Blood samples from the second patient were also sent, and rPCR results led to a diagnosis of *N. meningitidis* group C infection in both patients.

### Standard Culture-Based Tests versus rPCR

A total of 116 blood samples were tested with rPCR, and 107 blood samples were tested with culture (Table 3). Blood was positive for *N. meningitidis* in 104 (89.7%) of 116 samples tested with rPCR and in 26 (24.3%) of 107 samples tested with blood culture (odds ratio [OR] 27.0, 95% CI 12.1–61.2; p<0.0001). One culture sample was reported as contaminated with *Streptococcus viridans*.

**Table 3:** Distribution of rPCR and culture-based test results for *Neisseria meningitidis* for CSF and blood samples from a national register for molecular surveillance of invasive bacterial disease, Italy, 2007–2014*

Type of sample	No. samples/no. tested (%)			
	Positive by rPCR	Negative by rPCR	Not tested by rPCR	Total†
CSF				
Culture positive	33	0	0	33/90 (36.7)
Culture negative	55	2	0	57/90 (63.3)
Not tested with culture	72	0	0	0
Total	160/162 (98.8)	2/162 (1.2)	0	0
Blood				
Culture positive	16	0	10	26/107 (24.3)
Culture negative	37	10	34	81/107 (75.7)
Not tested with culture	51	2	0	0
Total	104/116 (89.7)	12/116 (10.3)	0	0
Total, CSF or blood				
Culture positive	49	0	10	59/197 (29.9)
Culture negative	92	12	34	138/197 (70.1)
Not tested with culture	123	2	0	0
Total	264/278 (95.0)	14/278 (5.0)	0	0

*A total of 162 CSF samples were tested with rPCR, and 90 were tested with culture-based methods. A total of 116 blood samples were tested with rPCR, and 107 were tested with culture-based methods. CSF, cerebrospinal fluid; rPCR, real-time PCR. †Proportion of samples that were positive, negative, or not tested with culture-based methods.

A total of 162 CSF samples were tested with rPCR, and 90 CSF samples were tested with culture (Table 3). CSF was positive in 160 (98.8%) of 162 samples tested with rPCR and in 33 (36.7%) of 90 samples tested with CSF culture (OR 138.1, 95% CI 30.7–862.6; p<0.0001). One culture sample was reported as contaminated with *S. epidermidis*. Overall, by considering both kinds of samples, rPCR was shown to be 3.28 times more sensitive than culture.

All 12 patients whose blood samples were negative by rPCR had CSF samples that tested positive by rPCR. Among the 81 patients whose samples tested negative by blood culture, CSF culture was not performed for 22 (27.2%); 18 (22.2%) had samples that tested positive by CSF culture and 41 (50.6%) had samples that tested negative by CSF culture.

The 2 patients whose CSF samples tested negative by rPCR had blood samples that tested positive by rPCR. Among the 57 patients whose samples tested negative by CSF culture, a blood culture was not performed for 14 (24.6%); a blood culture tested positive for *N. meningitidis* for 4 (7.0%) and negative for 39 (68.4%).

Overall (including CSF and blood samples), rPCR enabled a correct diagnosis of IMD in all (100%) patients. On the other hand, culture enabled a correct diagnosis in only 29 (42.0%) of 69 patients for whom blood and CSF cultures were performed at admission.

To better compare the sensitivity of rPCR versus culture, we evaluated samples collected at the same time and tested by using both methods. Of the 63 patients who had samples that were simultaneously tested with blood culture and rPCR on blood, 53 (84.1%) had samples that tested positive by rPCR, whereas 17 (26.9%) had samples that tested positive by culture (OR 14.3, 95% CI 5.5–38.2; p<0.0001); 45 (71.4%) had samples that tested negative by culture. One of the 17 samples that tested positive by culture was reported as contaminated. No sample found negative by rPCR was found positive by culture. Use of rPCR on blood was 3.12 times more sensitive than blood culture.

Eighty-eight patients had samples that were simultaneously tested with CSF culture and rPCR on CSF: 86 (97.7%) had samples that tested positive by rPCR, whereas 338 (37.5%) had samples that tested positive by culture (OR 71.6, 95% CI 15.7–451.1; p<0.0001); 54 (61.4%) had samples that tested negative by culture. One of the 33 samples that tested positive by culture was reported as contaminated. No sample found negative by rPCR was found positive by CSF culture, and rPCR on CSF was 2.61 times more sensitive than CSF culture.

Overall, *N. meningitidis* was identified only by rPCR in 36 of 63 blood samples and in 53 of 88 CSF samples. For enabling a laboratory diagnosis of IMD, rPCR was significantly more sensitive than culture (Cohen’s Kappa 0.59, OR 23.4, 95% CI 11.3–49.1; p<0.001).

## Discussion

Our analysis of the national register for molecular surveillance of bacterial disease in Italy showed that at least 2 main factors cause underestimation of IMD: misdiagnosis and insufficiently sensitive laboratory methods. In the register, 3 deceased patients had previously had a different disease diagnosis (i.e., SIDS, acute myeloid leukemia, sudden death); later, when biological samples were tested for *N. meningitidis* for other reasons (e.g., a legal trial or a secondary case), samples from the patients were found to be positive for the pathogen. The extent of misdiagnosis is difficult to quantify. Although misdiagnoses account for 1.4% in the national register, the actual percentage is probably much higher because only cases for which a clinical doubt occurred and samples were tested posthumously had a chance of being found positive for *N. meningitidis*. In the 3 cases described in this article, samples were retrieved and tested posthumously. However, in absence of those incidental situations, all 3 cases would have been misdiagnosed, thus contributing to the underestimation of IMD.

Among the 26 fatal cases, >40% were undiagnosed by standard culture-based methods, thus substantially contributing to the underestimation of IMD. In all undiagnosed cases, culture-based test results were either negative or not performed because sudden death attributable to *N. meningitidis* infection occurred before the patients were admitted to the hospital or upon their arrival at the emergency department. Whereas rPCR can be used for postmortem analysis of samples and enables diagnosis and serogrouping, culture-based methods are not useful in those situations; rPCR can be used with formalin-fixed tissue (*14*,*15*), as occurred with 2 of our patients, and even with bodies in advanced decomposition (*16*). Diagnoses of IMD is important for timely administration of prophylaxis to contacts and for limiting underestimation of cases. Therefore, rPCR should be considered as a fundamental tool. Moreover, molecular techniques offer the opportunity to identify the serogroup in culture-negative and fulminant cases. The ability to identify serogroups has important implications for vaccination programs. In fact, if fatalities were more often associated with a specific serogroup, a dedicated vaccination program could be planned. Moreover, molecular techniques enable the meningococcus to be molecularly characterized, which is important for planning and monitoring vaccination with subcapsular meningococcal vaccines.

We found that all tissues tested postmortem were positive for *N. meningitidis* by using rPCR. No specific kind of tissue seems to be better suited for diagnostic testing.

As for laboratory confirmation of IMD in nonfatal cases, current data confirm what has been shown previously about meningococcal (*5*,*17*) and pneumococcal (*18*,*19*) infections: rPCR is approximately 3 times more sensitive than culture in identifying meningococcal infection, regardless of the type of biologic sample used or the patients’ clinical signs and symptoms. Consequently, in countries (as in Italy) where most hospitals use only standard culture-based methods for diagnosis of invasive bacterial infections, incidence of IMD may be largely underestimated.

Testing with rPCR can enable etiologic diagnosis and serogrouping in culture-negative samples (*19*–*21*). Therefore, most countries have included rPCR techniques in addition to culture-based tests in surveillance programs. The results are encouraging: in developed areas, such as England or Wales, the number of diagnoses made has more than doubled with the use of rPCR because 58% of cases were confirmed by rPCR alone (*22*). Our study shows that in Italy, as in England and Wales, >50% of cases are confirmed by rPCR alone. The advantage is even greater in countries with fewer health resources, where laboratory results might be negatively influenced by inadequate transport and storage of samples (*23*). Testing with rPCR has the additional advantage of providing results rapidly, enabling speedy initiation of prophylaxis of contacts, thus preventing secondary cases.

Other underestimation factors undoubtedly exist, and underreporting is surely one of the most important (*24*). Clinicians must be made aware that, besides curing patients, identifying and reporting the bacterial etiology are important for enabling a better understanding of the epidemiology of meningococcal disease and implementation of appropriate public health interventions, such as vaccination programs or prophylaxis for contacts. Hospitals unable to offer local rPCR should be encouraged to duly and promptly collect samples for offsite testing.

In summary, IMD is largely underestimated in Italy because of misdiagnosis, limited use of molecularly based laboratory methods, and undernotification. Using molecular methods for diagnosis of IMD in all patients with clinical evidence that results in a suspicion of *N. meningitidis* infection and for postmortem diagnoses can help reduce underestimation of IMD.
